# Assessment of Hospital-Onset SARS-CoV-2 Infection Rates and Testing Practices in the US, 2020-2022

**DOI:** 10.1001/jamanetworkopen.2023.29441

**Published:** 2023-08-28

**Authors:** Kelly M. Hatfield, James Baggs, Alexander Maillis, Sarah Warner, John A. Jernigan, Sameer S. Kadri, Michael Klompas, Sujan C. Reddy

**Affiliations:** 1 Division of Healthcare Quality Promotion, Centers for Disease Control and Prevention, Atlanta, Georgia; 2 Critical Care Medicine Department, National Institutes of Health Clinical Center, Bethesda, Maryland; 3 Department of Population Medicine, Harvard Medical School and Harvard Pilgrim Healthcare Institute, Boston, Massachusetts; 4 Department of Medicine, Brigham and Women’s Hospital, Boston, Massachusetts

## Abstract

**Importance:**

Characterizing the scale and factors associated with hospital-onset SARS-CoV-2 infections could help inform hospital and public health policies regarding prevention and surveillance needs for these infections.

**Objective:**

To evaluate associations of hospital-onset SARS-CoV-2 infection rates with different periods of the COVID-19 pandemic, hospital characteristics, and testing practices.

**Design, Setting, and Participants:**

This cohort study of US hospitals reporting SARS-CoV-2 testing data in the PINC AI Healthcare Database COVID-19 special release files was conducted from July 2020 through June 2022. Data were collected from hospitals that reported at least 1 SARS-CoV-2 reverse transcription–polymerase chain reaction or antigen test during hospitalizations discharged that month. For each hospital-month where the hospital reported sufficient data, all hospitalizations discharged in that month were included in the cohort. SARS-CoV-2 viral tests and results reported in the microbiology files for all hospitalizations in the study period by discharge month were identified. Data analysis was conducted from September 2022 to March 2023.

**Exposure:**

Hospitalizations discharged in an included hospital-month.

**Main Outcomes and Measures:**

Multivariable generalized estimating equation negative-binomial regression models were used to assess associations of monthly rates of hospital-onset SARS-CoV-2 infections per 1000 patient-days (defined as a first positive SARS-CoV-2 test during after hospitalization day 7) with the phase of the pandemic (defined as the predominant SARS-CoV-2 variant in circulation), admission testing rates, and hospital characteristics (hospital bed size, teaching status, urban vs rural designation, Census region, and patient distribution variables).

**Results:**

A total of 5687 hospital-months from 288 distinct hospitals were included, which contributed 4 421 268 hospitalization records. Among 171 564 hospitalizations with a positive SARS-CoV-2 test, 7591 (4.4%) were found to be hospital onset and 6455 (3.8%) were indeterminate onset. The mean monthly hospital-onset infection rate per 1000 patient-days was 0.27 (95 CI, 0.26-0.29). Hospital-onset infections occurred in 2217 of 5687 hospital-months (39.0%). The monthly percentage of discharged patients tested for SARS-CoV-2 at admission varied; 1673 hospital-months (29.4%) had less than 25% of hospitalizations tested at admission; 2199 hospital-months (38.7%) had 25% to 50% of all hospitalizations tested, and 1815 hospital months (31.9%) had more than 50% of all hospitalizations tested at admission. Postadmission testing rates and community-onset infection rates increased with admission testing rates. In multivariable models restricted to hospital-months testing at least 25% of hospitalizations at admission, a 10% increase in community-onset SARS-CoV-2 infection rate was associated with a 178% increase in the hospital-onset infection rate (rate ratio, 2.78; 95% CI, 2.52-3.07). Additionally, the phase of the COVID-19 pandemic, the admission testing rate, Census region, and bed size were all significantly associated with hospital-onset SARS-CoV-2 infection rates.

**Conclusions and Relevance:**

In this cohort study of hospitals reporting SARS-CoV-2 infections, there was an increase of hospital-onset SARS-CoV-2 infections when community-onset infections were higher, indicating a need for ongoing and enhanced surveillance and prevention efforts to reduce in-hospital transmission of SARS-CoV-2 infections, particularly when community-incidence of SARS-CoV-2 infections is high.

## Key Points

**Question:**

How frequent are hospital-onset SARS-CoV-2 infections and what hospital characteristics are associated with rates of hospital-onset SARS-CoV-2 infections?

**Findings:**

This cohort study of 288 hospitals found that hospital-onset SARS-CoV-2 infections occurred at rates similar to those of other measured health care–associated infections; among 171 564 hospitalizations with a positive SARS-CoV-2 test, 7591 (4.4%) were found to be hospital onset and 6455 (3.8%) were indeterminate onset. In multivariable models, higher hospital-onset infection rates were associated with increases in community-onset SARS-CoV-2 infection rates, period of the COVID-19 pandemic, admission testing rate, Census region, and bed size.

**Meaning:**

Ongoing and enhanced surveillance and prevention efforts to reduce in-hospital transmission of SARS-CoV-2 infections are needed, particularly when community incidence of SARS-CoV-2 infections is high.

## Teaser

This cohort study analyzes rates of hospital-onset SARS-CoV-2 infections and their association with hospital-level characteristics and testing practices.

## Introduction

The COVID-19 pandemic has caused substantial disruption throughout the world since early 2020. Early in the pandemic, US hospitals adopted aggressive infection control measures and enhanced the use of personal protective equipment (eg, universal masking of staff and patients, testing of asymptomatic patients, and use of N95 respirators and eye protection) to prevent transmission of SARS-CoV-2, the virus that causes COVID-19.^1^

To date, hospital-onset SARS-CoV-2 infections have not been well described. Single health care system studies^2^ have demonstrated that the risk of health care–associated SARS-CoV-2 infections likely changed in accordance with community incidence. A study^3^ in the UK demonstrated that at least 14% (and up to 24%) of SARS-CoV-2 infections at 4 hospitals were found to be nosocomial. Furthermore, since new variants have emerged, single-center studies^4^ have described large-scale nosocomial outbreaks, even among fully vaccinated individuals. In December 2021, the Omicron variant of SARS-CoV-2 became the predominant strain circulating in the US.^5^ This variant is associated with higher levels of infectivity and likelihood of transmission, even among individuals with a completed initial vaccination series.^6^ Studies have shown^2,7^ that the surge of infections observed when the Omicron variant emerged was associated with a substantial increase in hospital-onset SARS-CoV-2 infections, suggesting a potential increased risk of acquiring SARS-CoV-2 infection during hospitalization.

The rates of hospital-onset COVID-19 among US hospitals and associated hospital-level factors are not well understood. Measuring hospital-onset infections across hospitals is complicated by potential variability in hospital testing practices throughout the pandemic. In this study, we aim to describe monthly rates of hospital-onset SARS-CoV-2 infection throughout the pandemic. Furthermore, we seek to assess whether hospital-onset infection rates are associated with hospital-level factors, hospital testing practices, or the predominant variant in circulation.

## Methods

This cohort study follows the Strengthening the Reporting of Observational Studies in Epidemiology (STROBE) reporting guideline^8^ and did not require institutional review board approval or informed consent because data were deidentified in accordance with 45 CFR § 46. We explored the incidence of hospital-onset SARS-CoV-2 infections using the PINC AI Healthcare Database (PHD), COVID-19 special release files.^9^ The files we extracted for this analysis were dated August 30, 2022. Formerly known as Premier, PHD is an all-payer, hospital-based, comprehensive electronic health care records database that stores data on more than 100 million inpatient admissions annually. PHD includes data on hospital characteristics, patient-level diagnostic codes, and other clinical information. For a subset of hospitals, PHD includes detailed microbiology laboratory data, including SARS-CoV-2 testing information. For our analyses, we used the PHD convenience sample to identify a cohort of hospitals that were reporting sufficient laboratory data (ie, at least 1 hospitalization included a SARS-CoV-2 real-time reverse transcription–polymerase chain reaction or antigen test reported in the laboratory data) from July 2020 through June 2022. For each hospital-month where the hospital reported sufficient data, we included all hospitalizations discharged from that hospital in that month into our cohort and identified all SARS-CoV-2 viral tests and results reported in the microbiology files for those hospitalizations.

### Testing Rates

To quantify hospital-level testing practices and determine the likelihood of case detection and the potential for misclassification, we calculated admission testing rates as the proportion of all hospitalizations with at least 1 admission test for each hospital-month. Admission testing was defined as a viral test for SARS-CoV-2 infection collected from 7 days before hospitalization to hospitalization day 3. Hospital-months were categorized according to monthly admission SARS-CoV-2 testing rates (ie, <25% of all hospitalizations tested, 25%-50% tested, and >50% tested). Postadmission testing rates were calculated for each hospital-month as number of SARS-CoV-2 tests per 1000 patient-days and stratified into 2 categories: early (tests collected on hospitalization days 4 through 7 per 1000 patient-days) and late (tests collected after hospital day 7 per 1000 patient-days).

### SARS-CoV-2 Infection Rates

Hospitalizations during which a patient had any positive SARS-CoV-2 test were categorized by the timing of the patient’s first positive test. Hospitalizations were categorized as community onset if the first positive SARS-CoV-2 test was collected in the admission period (ie, days −7 through 3 relative to admission), indeterminate onset if the first positive SARS-CoV-2 test was collected on day 4 through 7 of hospitalization, and hospital onset if the first positive SARS-CoV-2 test was collected after day 7 of hospitalization. Hospitalizations of patients with an* International Classification of Diseases, 10th Revision, Clinical Modification* (*ICD-10-CM*) diagnosis code for COVID-19 (U07.1) but no positive test recorded for SARS-CoV-2 during their hospitalization were categorized as unknown onset. The rates of hospitalizations with hospital-onset infections were calculated monthly overall and for each hospital per all hospitalizations, per at-risk hospitalizations, and per 1000 patient-days. For the purposes of this analysis, we considered all hospitalizations as at risk for community-onset infection and hospitalizations with a length of stay greater than 7 days as at risk for hospital-onset infection. To minimize misclassification bias of community-onset infections as hospital-onset infections because of potential incubation periods longer than 7 days, we used sensitivity analyses with 2 additional definitions of hospital-onset infections, defined by the first positive test after days 10 or 14 of hospitalization (with denominators for corresponding at-risk hospitalizations and all hospitalizations). Admission percentage positivity was calculated as the number of hospitalizations with a positive test at admission (ie, community-onset SARS-CoV-2 infection) per 100 hospitalizations tested at least once in the admission period.

### Factors Associated With Hospital-Onset SARS-CoV-2 Infection Rates

Infection rates and the admission percentage positivity were calculated monthly for each participating hospital. Hospital-onset infection rates were calculated as the number of hospital-onset SARS-CoV-2 infections per 1000 patient-days.

Hospital characteristics, including hospital bed size, teaching status, urban vs rural designation, and Census region, were ascertained from the PHD. For each month, we calculated variables to account for differences in patient makeup within each hospital. First, we calculated the percentage of discharges from each hospital among patients aged 65 years and older to describe potentially important differences in age distributions by hospital. Second, we calculated the percentage of discharges from each hospital among patients with a race and ethnicity listed as non-White non-Hispanic in the database to describe differences in the distributions of patient race and ethnicity. Race in the PHD were identified from the UB-04 form as Asian, Black, White, and other race (defined by PHD to ensure that the data conformed to HIPPA and other regulatory requirements); a separate variable indicated Hispanic ethnicity designation. Hospital months were categorized into 3 pandemic periods on the basis of the predominant SARS-CoV-2 variant in circulation in the US and included the pre-Delta period for discharges occurring in months between July 2020 and May 2021, Delta period for discharges occurring in June 2021 through December 2021, and Omicron period for discharges in January 2022 or later.^5^

### Statistical Analysis

Hospital characteristics were compared across the 3 admission testing rate categories using χ^2^ tests of independence for categorical variables and generalized linear models for continuous variables. Generalized linear models were used to compare mean postadmission testing rates, infection rates, admission percentage positivity, and mean monthly hospital-level community-onset and hospital-onset SARS-CoV-2 infection rates across the 3 admission testing rate categories.

Multivariable generalized estimating equation negative-binomial regression models were used to assess associations of monthly facility-level hospital-onset SARS-CoV-2 infection rates with predominant SARS-CoV-2 variant period, admission testing rates, the proportion of all hospitalizations with a community-onset SARS-CoV-2 infection, hospital bed size, teaching status, urban vs rural designation, Census region, and the patient distribution variables. Generalized estimating equation models accounted for clustering of data within hospitals using an exchangeable correlation structure. We exponentiated the coefficients from this regression model to develop rate ratio (RR) estimates with 95% CIs. In adjusted hospital-level models, we excluded hospital-months where less than 25% of admissions were tested for SARS-CoV-2 because of the high risk for misclassification in these hospitals (ie, community-onset cases were not identified until later in the admission) and for differential case finding (ie, that lower admission testing was associated with lower postadmission testing rates, potentially artificially reducing the hospital-onset infection rate). A 2-sided *P* < .05 was considered statistically significant. Analyses were conducted using SAS statistical software version 9.4 (SAS Institute). Data analysis was conducted from September 2022 to March 2023.

## Results

We included 5687 hospital-months from 288 distinct hospitals between July 2020 and June 2022 in our study cohort; 156 hospitals (54.2%) reported data in all 24 months of the study period. Characteristics from hospital-months are described in Table 1. More than one-half of the hospital-months were from hospitals with fewer than 200 beds (1744 [30.7%] from hospitals with 0-99 beds, and 1466 hospital-months [25.8%] from hospitals with 100-199 beds). The hospitals were predominantly located in the South (3146 hospital-months [55.3%]), predominantly nonteaching (4211 hospital-months [74.0%), and from urban areas (3747 hospital-months [65.9%]). The pre-Delta period contributed the most hospital-months (2757 hospital-months [48.5%]), followed by the Delta period (1672 hospital-months [29.4%]), and the Omicron period (1258 hospital-months [22.1%]). From these hospital-months, data from 4 421 268 hospitalizations were ascertained and aggregated.

**Table 1.** SARS-CoV-2 Testing and Infection Rates Stratified by Proportion of All Hospitalizations With Admission Testing

Hospital characteristics	Hospital-months, No. (%), by proportion of all hospitalizations tested for SARS-CoV-2 at admission (N = 5687)				*P* value^a^
	Overall (N = 5687)	<25% (n = 1673)	25%-50% (n =2199)	>50% (n = 1815)	
Distinct hospitals, No.^b^	288	211	227	161	Not applicable
Bed size					
0-99	1744 (30.7)	591 (35.3)	439 (20.0)	714 (39.3)	<.001
100-199	1466 (25.8)	398 (23.8)	661 (30.1)	407 (22.4)	
200-299	865 (15.2)	218 (13.0)	402 (18.3)	245 (13.5)	
300-399	675 (11.9)	165 (9.9)	305 (13.9)	205 (11.3)	
400-499	249 (4.4)	117 (7.0)	96 (4.4)	36 (2.0)	
≥500	688 (12.1)	184 (11.0)	296 (13.5)	208 (11.5)	
Census region^c^					
Midwest	1656 (29.1)	582 (34.8)	674 (30.7)	400 (22.0)	<.001
Northeast	561 (9.9)	128 (7.7)	114 (5.2)	319 (17.6)	
South	3146 (55.3)	902 (53.9)	1291 (58.7)	953 (52.5)	
West	324 (5.7)	61 (3.6)	120 (5.5)	143 (7.9)	
Teaching status					
Teaching	1476 (26.0)	396 (23.7)	611 (27.8)	469 (25.8)	.02
Nonteaching	4211 (74.0)	1277 (76.3)	1588 (72.2)	1346 (74.2)	
Urban vs rural designation					
Urban	3747 (65.9)	1089 (65.1)	1571 (71.4)	1087 (59.9)	<.001
Rural	1940 (34.1)	584 (34.9)	682 (31)	728 (40.1)	
Patient distributions, mean (SD)					
Discharges with patients aged ≥65 y	45.6 (16.2)	44.4 (16.2)	43.3 (15.1)	49.5 (16.9)	<.001
% Discharges with race and ethnicity listed as non-White, non-Hispanic	28.7 (21.1)	23.6 (18.5)	31.3 (20.6)	30.1 (23.1)	<.001
Pandemic period					
Pre-Delta (July 2020 to May 2021)	2757 (48.5)	657 (39.3)	1139 (51.8)	961 (52.9)	<.001
Delta (June 2021 to December 2021)	1672 (29.4)	472 (28.2)	668 (30.4)	532 (29.3)	
Omicron (January 2022 to July 2022)	1258 (22.1)	544 (32.5)	392 (17.8)	322 (17.7)	

^a^ Comparison of categorical characteristics across groups was calculated using χ^2^ tests for independence; comparison of continuous variables was calculated using generalized linear models. *P* values comparing homogeneity in frequency or means among the 3 admission testing rates were calculated by χ^2^ or generalized linear models.

^b^ Hospitals can be in more than 1 group due to differences in monthly testing practices.

^c^ States by US census region included the Midwest (Illinois, Indiana, Iowa, Kansas, Michigan, Minnesota, Missouri, Nebraska, North Dakota, Ohio, South Dakota, and Wisconsin); Northeast (Connecticut, Maine, Massachusetts, New Hampshire, New Jersey, New York, Pennsylvania, Rhode Island, and Vermont); South (Alabama, Arkansas, Delaware, Florida, Georgia, Kentucky, Louisiana, Maryland, Mississippi, North Carolina, Oklahoma, South Carolina, Tennessee, Texas, Virginia, Washington DC, and West Virginia); and West (Alaska, Arizona, California, Colorado, Hawaii, Idaho, New Mexico, Montana, Nevada, Oregon, Utah, Washington, and Wyoming ).

### Admission Testing Rates

Among 5687 hospital months, 1673 (29.4%) had less than 25% of all hospitalizations tested for SARS-CoV-2 infection at admission, 2199 (38.7%) had 25% to 50% of all hospitalizations tested at admission, and 1815 (31.9%) had more than 50% of all hospitalizations tested at admission. Hospital characteristics varied significantly by admission testing rates (Table 1). Overall, admission testing was conducted in 1 642 632 (37.2%) of all hospitalizations. The mean early and late postadmission testing rates increased as the proportion of hospitalizations with admission testing increased (Table 2). Aggregate monthly postadmission testing rates stratified by admission testing categorization are shown in eFigure 1 in Supplement 1).

**Table 2.** Mean Monthly Testing and Infection Rates Stratified by Proportion of All Hospitalizations Tested for SARS-CoV-2 at Admission

SARS-CoV-2 onset infection or testing rate	Proportion of all hospitalizations tested for SARS-CoV-2 at admission				*P *value^a^
	Overall	<25%	25%-50%	>50%	
Hospital-months, No.	5687	1673	2199	1815	NA
Testing rate, mean (95% CI)					
Early postadmission testing^b^	40.5 (39.3-41.7)	24.2 (22.2-26.2)	42.2 (40.8-43.7)	53.4 (50.6-56.2)	<.001
Late postadmission testing^c^	44.3 (42.6-46.1)	23.0 (20.5-25.4)	47.2 (44.7-49.7)	60.6 (56.9-64.3)	<.001
Admission percentage positivity^d^	10.9 (10.6-11.2)	11.1 (10.4-11.9)	10.8 (10.4-11.2)	10.9 (10.4-11.4)	.46
Infection rate per 1000 patient-days (95% CI)					
Community^e^	4.15 (4.02-4.28)	1.29 (1.20-1.38)	4.16 (4.00-4.33)	6.76 (6.45-7.08)	<.001
Indeterminate ^f^	0.30 (0.28-0.32)	0.18 (0.14-0.22)	0.33 (0.30-0.35)	0.37 (0.32-0.42)	<.001
Hopsital^g^	0.27 (0.26-0.29)	0.16 (0.13-0.20)	0.32 (0.30-0.35)	0.32 (0.29-0.35)	.003
Unknown^h^	5.48 (5.30-5.66)	6.23 (5.83-6.63)	5.48 (5.21-5.74)	4.78 (4.49-5.07)	<.001

Abbreviation: NA, not applicable.

^a^ *P* values were calculated using generalized linear models.

^b^ Early postadmission testing was defined as the number of tests occurring on hospitalization days 4-7 per 1000 hospital-days.

^c^ Late postadmission testing was defined as the number of tests occurring after hospitalization day 7 per 1000 hospital-days.

^d^ Admission percentage positivity was defined as the number of hospitalizations with first positive SARS-CoV-2 test before day 4 of hospitalization per 100 hospitalizations tested for SARS-CoV-2 before day 4 of hospitalization.

^e^ Community-onset rate was defined as the number of hospitalizations with first positive SARS-CoV-2 test before day 4 of hospitalization per 100 hospitalizations.

^f^ Indeterminate-onset rate was defined as the number of hospitalizations with first positive SARS-CoV-2 test on day 4 through 7 of hospitalization per 1000 patient-days.

^g^ Hospital-onset rate was defined as the number of hospitalizations with first positive SARS-CoV-2 test after day 7 of hospitalization per 1000 patient-days.

^h^ Unknown-onset rate was defined as the number of hospitalizations with an* International Classification of Diseases, Tenth Revision, Clinical Modification ICD-10-CM* diagnosis code for COVID-19 (U07.1) but no recorded positive SARS-CoV-2 test per 100 hospitalizations.

### SARS-CoV-2 Infection Rates

Among all 4 421 268 hospitalizations, 171 564 (3.9%) had at least 1 positive SARS-CoV-2 test; 157 518 (3.6%) were categorized as community-onset SARS-CoV-2 infections, 6455 (0.1%) were indeterminate-onset, and 7591 (0.2%) were hospital-onset. Among all 171 564 hospitalizations with positive SARS-CoV-2 tests, 157 518 (91.8%) were categorized as community-onset, 6455 (3.8%) as indeterminate-onset, and 7591 (4.4%) as hospital-onset. Taken together, indeterminate-onset and hospital-onset infections account for 14 046 (8.2%) of all hospitalizations with a positive SARS-CoV-2 test.

Most hospitalizations with a positive SARS-CoV-2 test (135 256 hospitalizations [78.8%]) had the first positive test on day 1 of hospitalization. An additional 187 488 hospitalizations (4.4%) had an *ICD-10-CM* diagnosis code for COVID-19 (U07.1) but no positive test recorded for SARS-CoV-2 during the hospitalization and were categorized as unknown onset. Characteristics for hospitalizations by SARS-CoV-2 infection status are described in eTable 1 in Supplement 1.

The mean hospital-month admission percentage positivity was 10.9% (95% CI, 10.6%-11.2%) and did not vary by admission testing rates (Table 2). The mean community-onset SARS-CoV-2 infection rates varied over time (eFigure 2 in Supplement 1) and increased as admission testing increased (Table 2). The aggregate percentage of all hospitalizations with unknown-onset SARS-CoV-2 infections (ie, hospitalizations with an *ICD-10-CM* diagnosis code but without a SARS-CoV-2 positive test) varied over time (eFigure 3 in Supplement 1). The mean monthly rate of hospitalizations with unknown-onset SARS-CoV-2 infection rate was highest in the hospital-months with less than 25% of hospitalizations tested at admission for SARS-CoV-2 (6.2%; 95% CI, 5.8%-6.6%) compared with 25% to 50% (5.5%; 95% CI, 5.2%-5.7%) and greater than 50% (4.8%; 95% CI, 4.5%-5.1%; *P* < .001) (Table 2).

### Hospital-Onset and Indeterminate-Onset SARS-CoV-2 Infections

Throughout the entire study period, we identified 7591 hospitalizations with hospital-onset SARS-CoV-2 infection and 6455 hospitalizations with indeterminate-onset SARS-CoV-2 infection among 21 923 622 patient-days, for an aggregate overall SARS-CoV-2 infection rate of 0.35 hospital-onset infections per 1000 patient-days and 0.29 indeterminate-onset infections per 1000 patient-days. The mean monthly hospital-level rate was 0.27 hospital-onset SARS-CoV-2 infections per 1000 patient-days (95% CI, 0.26 0.29) and 0.30 indeterminate-onset SARS-CoV-2 infections per 1000 patient-days (95% CI, 0.28-0.32). (Table 2). Among 5687 hospital-months included in our study, 2217 (39.0%) had at least 1 hospital-onset SARS-CoV-2 infection. However, in the 1679 hospital-months where less than 1% of hospitalizations had community-onset SARS-CoV-2 infections (ie, a positive test at admission), only 274 (16.3%) had at least 1 hospital-onset SARS-CoV-2 infection (eTable 2 in Supplement 1).

**Figure 1.**
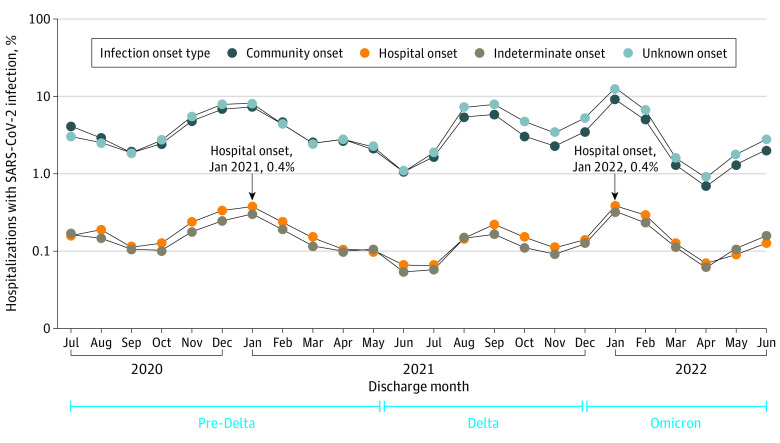
Aggregate Monthly Percentage of All Hospitalizations With Community-Onset, Indeterminate-Onset, Hospital-Onset, and Unknown-Onset SARS-CoV-2 Infections Community-onset includes hospitalizations with first positive SARS-CoV-2 test at admission (−7 to 3 days relative to admission); indeterminate-onset includes hospitalizations with first positive test on hospitalization days 4 to 7 relative to admission; hospital-onset includes hospitalizations with first positive test after day 7 relative to admission; and unknown-onset includes hospitalizations with an* International Classification of Diseases, Tenth Revision, Clinical Modification *diagnosis code for COVID-19 (U07.1) but no positive SARS-CoV-2 test recorded during hospitalization.

Overall, the monthly proportion of all hospitalizations with hospital-onset SARS-CoV-2 infection peaked in January 2021 (0.4%) and January 2022 (0.4%), which was concomitant with peaks in community-onset, indeterminate-onset, and unknown-onset infections (Figure 1). Regardless of the hospital-onset definition, aggregate proportions of at-risk hospitalizations with hospital-onset SARS-CoV-2 infection were similar, and the proportion per all hospitalizations and infection rates followed similar trends (eFigure 4 in Supplement 1).

The mean hospital-onset infection rate in months with less than 25% of hospitalizations with admission testing for SARS-CoV-2 was 0.16 infections per 1000 patient-days (95% CI, 0.13-0.20). Conversely, the mean hospital-onset infection rate was 0.32 infections per 1000 patient-days (95% CI, 0.30-0.35) in hospital-months with 25% to 50% of hospitalizations tested at admission and 0.32 infections per 1000 patient-days (95% CI, 0.29-0.35) in hospital-months with more than 50% of hospitalizations tested at admission. (*P* = .003) (Table 2). In hospital-months with greater than 5% of all hospitalizations having a community-onset SARS-CoV-2 infection, the mean hospital-onset SARS-CoV-2 infection rate per 1000 patient days was lowest in hospital-months that tested less than 25% of hospitalizations at admission (rate per 1000 patient days, 0.38; 95% CI, 0.23-0.53); hospitals that tested more than 50% of hospitalizations at admission had a lower mean rate (rate per 1000 patient days, 0.46; 95% CI, 0.40-0.51) than hospitals that tested 25% to 50% of hospitalizations at admission (rate per 1000 patient-days 0.52; 95% CI, 0.46-0.59) (eTable 2 in Supplement 1). Aggregate hospital-onset SARS-CoV-2 infection rates were also lowest for hospitals that tested less than 25% of admissions each month (eFigure 5 in Supplement 1. Indeterminate-onset SARS-CoV-2 infections were also lowest in hospital-months with less than 25% of hospitalizations tested at admission for SARS-CoV-2 (0.18 infections per 1000 patient-days; 95% CI, 0.14-0.22) compared with hospital-months with 25% to 50% of hospitalizations tested at admission (0.33 infections per 1000 patient-days; 95% CI, 0.30-0.35), and hospital-months with more than 50% of hospitalizations tested at admission (0.37 infections per 1000 patient-days; 95% CI, 0.32-0.42).

### Factors Associated With Hospital-Level SARS-CoV-2 Hospital-Onset Infection Rates

**Figure 2.**
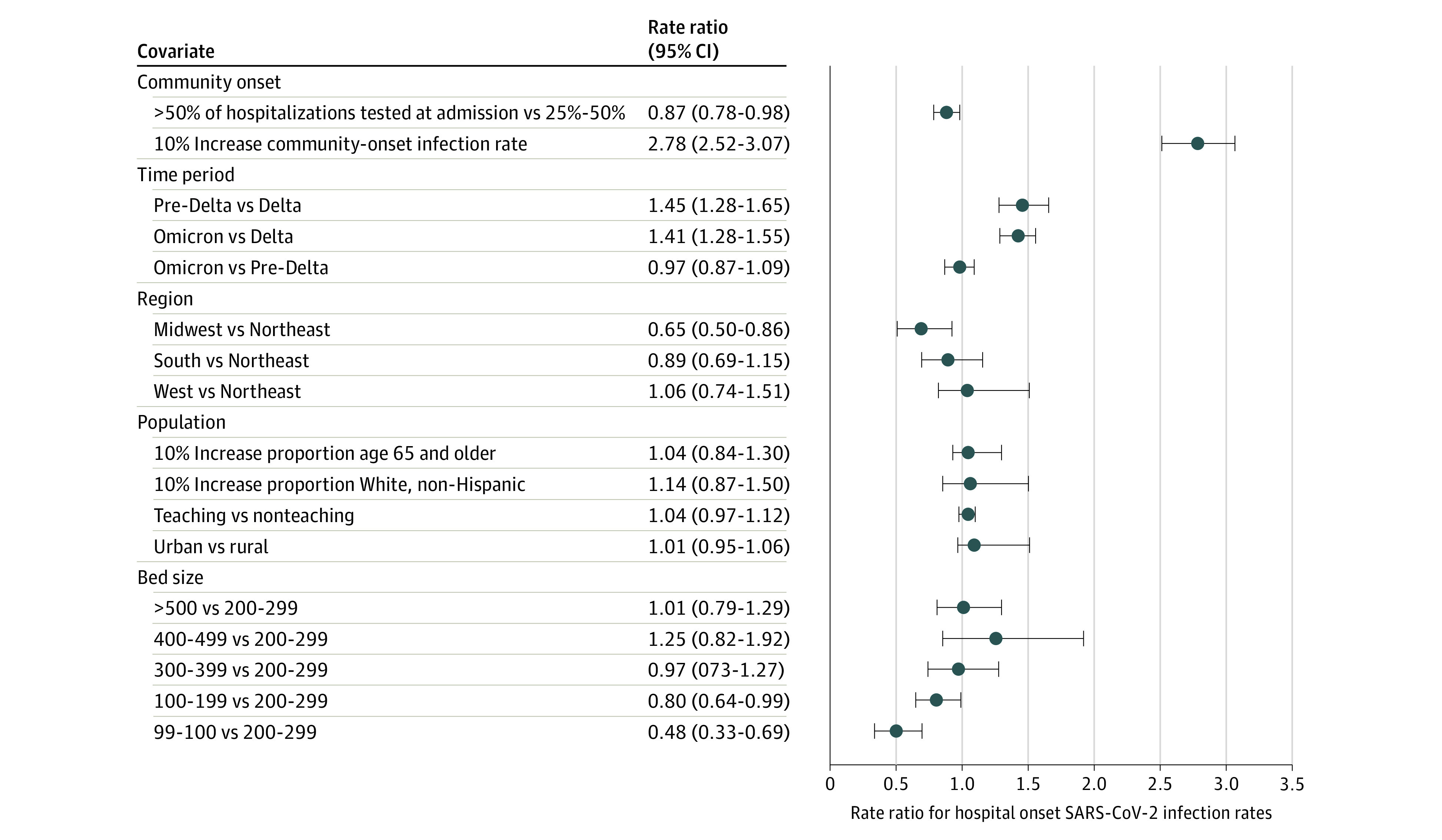
Association of Hospital-Level Factors with Hospital-Onset SARS-CoV-2 Infections The figure shows incidence rate ratios from multivariable generalized estimating equation negative-binomial regression models assessing hospital-level factors and their association with monthly hospital-onset SARS-CoV-2 infection rates per 1000 patient-days, among 4020 hospital-months where greater than 25% of admissions were tested for SARS-CoV-2 between July 2020 and June 2022. Dots denote means and bars denote 95% CIs. Data were collected from the PINC A1 Healthcare Database.

In multivariable models that included all hospital-months where at least 25% of admissions were tested for SARS-CoV-2 infection, we found that the proportion of hospitalizations with community-onset infections, predominant variant period, admission testing rate, bed size, and the geographic Census region of the hospital were all significantly associated with hospital-onset SARS-CoV-2 infection rates (Figure 2). A 10% increase in the proportion of all hospitalizations with community-onset SARS-CoV-2 infections was associated with a 178% increase in the rate of hospital-onset SARS-CoV-2 infections (RR, 2.78; 95% CI, 2.52-3.07). In the adjusted models, hospitals that tested more than 50% of admissions were associated with 13% decrease in hospital-onset SARS-CoV-2 infection rates (RR, 0.87; 95% CI, 0.78-0.98) compared with hospitals testing 25% to 50% of admissions. The pre-Delta and Omicron periods had higher adjusted rates of hospital-onset SARS-CoV-2 infections compared with the Delta period (RR for pre-Delta, 1.45 [95% CI, 1.28-1.65] and RR for Omicron, 1.41 [95% CI, 1.28-1.55]). Adjusted hospital-onset infection rates in the Omicron period were not significantly different from the pre-Delta period (RR, 0.97; 95% CI, 0.87-1.09). Hospitals in the 2 smallest bed size categories had lower rates of hospital-onset SARS-CoV-2 infections compared with those with 200 to 299 beds; however, larger hospitals did not have significantly different rates (Figure 2). Hospital-onset SARS-CoV-2 infection rates varied by geographic Census region of the hospital and were significantly lower in the Midwest compared with the Northeast (Figure 2). Other hospital characteristics, including population demographics, urban vs rural location, and teaching status, were not significantly associated with hospital-onset SARS-CoV-2 infection rates.

## Discussion

In this cohort study, we identified hospital-onset SARS-CoV-2 infections in 0.2% of all hospitalizations in our study period, which nearly doubled to 0.4% of all hospitalizations in peak months. The proportion of hospitalizations with hospital-onset SARS-CoV-2 infection is comparable to point prevalence estimates of several other health care-associated infections, including urinary tract infections (0.3% of all patients) and bloodstream infections (0.4%).^10^ Preventing morbidity and mortality from health care-associated infections is a top priority for the US Department of Health and Human Services and is included among the objectives for Healthy People 2030.^11^ The frequency of hospital-onset SARS-CoV-2 infections emphasizes the need for continued surveillance and attention to infection prevention practices related to SARS-CoV-2 transmission within a hospital setting.

Hospital-level testing practices were associated with the observed rates of community-onset and hospital-onset SARS-CoV-2 infections. Hospital-level SARS-CoV-2 admission testing rates were positively associated with postadmission SARS-CoV-2 testing rates, even during periods of high community incidence, meaning that hospitals that were less likely to test at admission were also less likely to test for SARS-CoV-2 throughout the hospitalization stay. Hospitals with less frequent testing after admission may have had artificially lower observed hospital-onset SARS-CoV-2 infection rates due to poor data capture because of undertesting. Multivariable adjusted models that excluded hospital-months with the lowest levels of admission testing and accounted for pandemic period and community-onset infection rates suggested that the highest levels of admission testing were associated with lower rates of hospital-onset SARS-CoV-2 infection, which may be because admission testing might minimize misclassification of community-onset cases as hospital-onset cases. Alternatively, admission testing might serve as an infection control strategy to prevent hospital-onset infections by identifying potentially contagious patients upon arrival. Additionally, high rates of admission testing could be a proxy indicator for more intense infection-control programs that include additional interventions to prevent hospital-onset cases, such as better adherence to universal masking, more attention to ventilation, and increased use of respirators for both health care worker protection and source control. Furthermore, comparing SARS-CoV-2 infection rates without accounting for variable testing density and practices between facilities and over time may be inappropriate.

Multivariable models among facilities testing at least 25% of the population suggested that hospital-onset SARS-CoV-2 infection rates were associated with community-onset infection rates. These associations demonstrate that the risk for hospital-onset SARS-CoV-2 infections is likely higher during periods of high infection rates in the community potentially due to higher importation rates from patients (including those who may be asymptomatic or test negative with latent infection at admission), visitors, and staff. Previous studies^12^ have shown that periods of high COVID-19 caseload surge exacerbate stress on hospital systems and lead to poorer outcomes among patients with COVID-19. Whether our observed association of increased community-onset infection SARS-CoV-2 infections with increased rates of hospital-onset infections was due to increased importation into the facility, crowding, lapses in infection control practices when hospital systems are stressed, or some combination of these factors is not clear from our study.

Our models also elucidated differences in hospital-onset SARS-CoV-2 infection rates by the pandemic period. The pandemic period served as a surrogate for both the predominant variant in circulation throughout the US and temporally associated changes in other factors. For example, levels of immunity (due to vaccination or prior infection) were higher in the Delta and Omicron periods compared with the pre-Delta period. However, the Omicron period had higher hospital-onset SARS-CoV-2 infection rates compared with the Delta period, potentially due to Omicron variant characteristics such as greater transmissibility and immune escape that, in turn, led to months of higher true community incidence.^2^ Studies^2,13,14^ have observed higher rates of hospital-onset SARS-CoV-2 infections associated with Omicron, which is potentially attributable to greater transmissibility, higher community incidence rates, or both. However, our study showed nonsignificant differences during the pre-Delta period and the Omicron period. These differences may be because our models reflected a longer time period (including both surge and nonsurge months), or due to factors, such as changes in infection control practices over time, timing of vaccination in both patients and health care workers, or differences in the hospitals included in each cohort.

We measured hospital-onset SARS-CoV-2 infections as those that occurred after day 7 of hospitalization. This measurement assumes that infections after day 7 were not present on admission and was selected to be longer than the approximate incubation period of SARS-CoV-2.^15^ However, it is likely that we undercounted true hospital-onset infections because the indeterminate-onset infections (ie, those occurring on days 4 through 7 of hospitalization) likely captured a mixture of both community-onset and hospital-onset infections. Taken together, indeterminate-onset and hospital-onset infections accounted for nearly 1 in 12 SARS-CoV-2 hospitalizations.

The Centers for Disease Control and Prevention recommends engineering and administrative controls, source control, transmission-based precautions, and other interventions to prevent transmission of SARS-CoV-2 in hospital settings.^16^ This study further reinforces the Centers for Disease Control and Prevention’s recommendation to modify those strategies when community transmission increases or when there is suspected transmission in a facility,^16^ because we identified community-onset infection rates as being significantly associated with hospital-onset infection rates. Limited single-center epidemiologic studies^17^ have proposed hospital-onset SARS-CoV-2 infection prevention strategies (eg, health care worker testing programs or universal N95 mask usage) as effective techniques to interrupt transmission in hospital-based clusters. Modeling studies^18^ have also suggested contact tracing with testing for SARS-CoV-2 at admission and on a serial basis thereafter as a highly effective tool in reducing transmission in a hospital setting. Our study showed that admission testing might also be considered a prevention strategy to reduce the number of hospital-onset SARS-CoV-2 infections within a hospital. Our models indicated that hospital-onset SARS-CoV-2 infection rates were lower among hospitals with greater than 50% of hospitalizations tested for SARS-CoV-2 infection at admission vs those with 25% to 50% tested. For admission testing to reduce transmission of SARS-CoV-2 within a hospital setting, early identification must be coupled with appropriate isolation and infection control precautions. SARS-CoV-2 characteristics (eg, proportion of hospitalizations with community-onset infections and predominant variant phase of the pandemic) were also associated with hospital-onset infection rates in multivariable models. Thus, prevention strategies should be enhanced during peak community incidence periods.

### Limitations

This study is subject to limitations. First, hospital-onset SARS-CoV-2 infection rates were determined using the day of the first positive test, and there was the potential for various types of misclassifications of infection. We selected a 7-day cutoff for hospital-onset categorization, consistent with various definitions used in other studies,^15^ but could not couple it with additional epidemiologic, clinical, or genetic testing information to accurately ascertain infection onset.^19^ We also defined our hospital-onset rate using several different denominators and found similar aggregate monthly rates. Second, because our study spanned several different variant predominance periods and phases of the pandemic, the epidemiologic characteristics of SARS-CoV-2 infections may have changed over the study period. For example, infections caused by the Omicron variant likely have a shorter incubation period than infections with previous variants.^20^ We attempted to address this difference in our multivariable models by incorporating a variable representing the national predominance period, but additional regional variation also occurred. Third, we were unable to combine a measure of community incidence that was independent of hospital testing practice due to a lack of specific information on hospital location in the data set. Our community incidence measure was derived on hospitalized patients only and did not represent incidence in the community, including among staff or other visitors in the hospital. Although there were some differences in community incidence between pandemic phases (eg, some months of very high incidence of community-onset infections during the Omicron phase) these were mixed with months of low incidence, such that pandemic phase would not meaningfully control for community incidence. Fourth, our analysis measures could not incorporate testing that occurred at home or prior to hospital admission. We identified nearly one-half of hospitalizations with an *ICD-10-CM* diagnosis code for COVID-19 that did not have a positive test during hospitalization; therefore, we cannot ascertain the timing of the onset of these infections. Fifth, despite our multivariable models excluding hospital months with less than 25% of hospitalizations tested at admission to reduce misclassification bias from a lack of admission testing, high proportion of infections with unknown onset, and the reduced rate of postadmission testing, variability in testing practices may still have influenced our results. Sixth, variability in hospital practices regarding the type of SARS-CoV-2 diagnostic tests used was not assessed. Higher rates of testing for community-onset cases may not have the same effect on preventing community-onset cases being misclassified as hospital-onset or interrupting transmission if tests with lower sensitivity in detecting asymptomatic cases (eg, antigen tests) are used. Seventh, we could not ascertain how hospital-onset SARS-CoV-2 infection rates may have been affected by unmeasured facility-level factors (eg, hospital-level implementation of infection control activities, visitation policies, staffing shortages and associated mitigation strategies, use of single vs shared rooms for patients, use of respirators or other face coverings for non–COVID-19 patient care, and the quality of ventilation) or the importance of those factors relative to the predominant variant in circulation or vaccination status.

## Conclusions

In this study, hospital-onset SARS-CoV-2 infections occurred in hospitals at similar rates as other health care–associated infections, particularly in peaks of community-onset transmission. This finding raises further questions about the preventability of these infections in hospital settings. Continued work is needed to determine optimal tools for the prevention of transmission of SARS-CoV-2 infection in a hospital setting and to ensure that these strategies are pragmatic and robust when community cases increase and put stress on the health care system.

## Notes

Supplement 1. **eFigure 1.** Overall Post-Admission Testing Rates, Stratified by the Proportion of Hospitalizations Tested at Admission **eTable 1.** Hospitalization Characteristics of Individuals Stratified by SARS-CoV-2 Infection Status **eFigure 2.** Aggregate Percent of All Hospitalizations With Community-Onset SARS-CoV-2 Infection, Stratified by the Proportion of Hospitalizations Tested at Admission **eFigure 3.** Aggregate Percent of All Hospitalizations With Unknown-Onset SARS-CoV-2 Infection, Stratified by the Proportion of Hospitalizations Tested at Admission **eTable 2.** Hospital-Onset SARS-CoV-2 Infections, Stratified by Community-Onset SARS-CoV-2 Infection Rates **eFigure 4.** Aggregate Hospital-Onset SARS-CoV-2 Infection Rates Using 3 Definitions **eFigure 5.** Aggregate Hospital-Onset SARS-CoV-2 Infection Rates per 1000 Patient-Days Stratified by the Proportion of Hospitalizations Tested at Admission

Supplement 2.
Data Sharing Statement
