# Supplementary material for: Assessment of Hospital-Onset SARS-CoV-2 Infection Rates and Testing Practices in the US, 2020-2022
Source: JAMA Netw Open. 2023 Aug 28;6(8):e2329441. doi: 10.1001/jamanetworkopen.2023.29441 (PMC10463096; doi:10.1001/jamanetworkopen.2023.29441)
Supplement: Supplement 1. — eFigure 1. Overall Post-Admission Testing Rates, Stratified by the Proportion of Hospitalizations Tested at Admission eTable 1. Hospitalization Characteristics of Individuals Stratified by SARS-CoV-2 Infection Status eFigure 2. Aggregate Percent of All Hospitalizations With Community-Onset SARS-CoV-2 Infection, Stratified by the Proportion of Hospitalizations Tested at Admission eFigure 3. Aggregate Percent of All Hospitalizations With Unknown-Onset SARS-CoV-2 Infection, Stratified by the Proportion of Hospitalizations Tested at Admission eTable 2. Hospital-Onset SARS-CoV-2 Infections, Stratified by Community-Onset SARS-CoV-2 Infection Rates eFigure 4. Aggregate Hospital-Onset SARS-CoV-2 Infection Rates Using 3 Definitions eFigure 5. Aggregate Hospital-Onset SARS-CoV-2 Infection Rates per 1000 Patient-Days Stratified by the Proportion of Hospitalizations Tested at Admission [file jamanetwopen-e2329441-s001.pdf]

Supplemental Online Content

Hatfield KM, Baggs J, Maillis A, et al. Assessment of hospital-onset SARS-CoV-2 infection rates and testing practices in the US. *JAMA Netw Open*. 2023;6(8):e2329441. doi:10.1001/jamanetworkopen.2023.29441

eFigure 1. Overall Post-Admission Testing Rates, Stratified by the Proportion of Hospitalizations Screened at Admission

eTable 1. Hospitalization Characteristics of Individuals Stratified by SARS-CoV-2 Infection Status

eFigure 2. Aggregate Percent of All Hospitalizations With Community-Onset SARS-CoV-2 Infection, Stratified by the Proportion of Hospitalizations Screened at Admission

eFigure 3. Aggregate Percent of All Hospitalizations With Unknown-Onset SARS-CoV-2 Infection, Stratified by the Proportion of Hospitalizations Screened at Admission

eTable 2. Hospital-Onset SARS-CoV-2 Infections, Stratified by Community-Onset SARS-CoV-2 Infection Rates

eFigure 4. Aggregate Hospital-Onset SARS-CoV-2 Infection Rates Using 3 Definitions

eFigure 5. Aggregate Hospital-Onset SARS-CoV-2 Infection Rates per 1000 Patient-Days Stratified by the Proportion of Hospitalizations Screened at Admission

This supplemental material has been provided by the authors to give readers additional information about their work.

© 2023 Hatfield KM et al. *JAMA Network Open.*

eFigure 1. Overall Post-Admission Testing Rates, Stratified by the Proportion of Hospitalizations Screened at Admission.

(a)Early post-admission testing (i.e., number of tests on days 4-7 of admission per 1,000 patient-days).

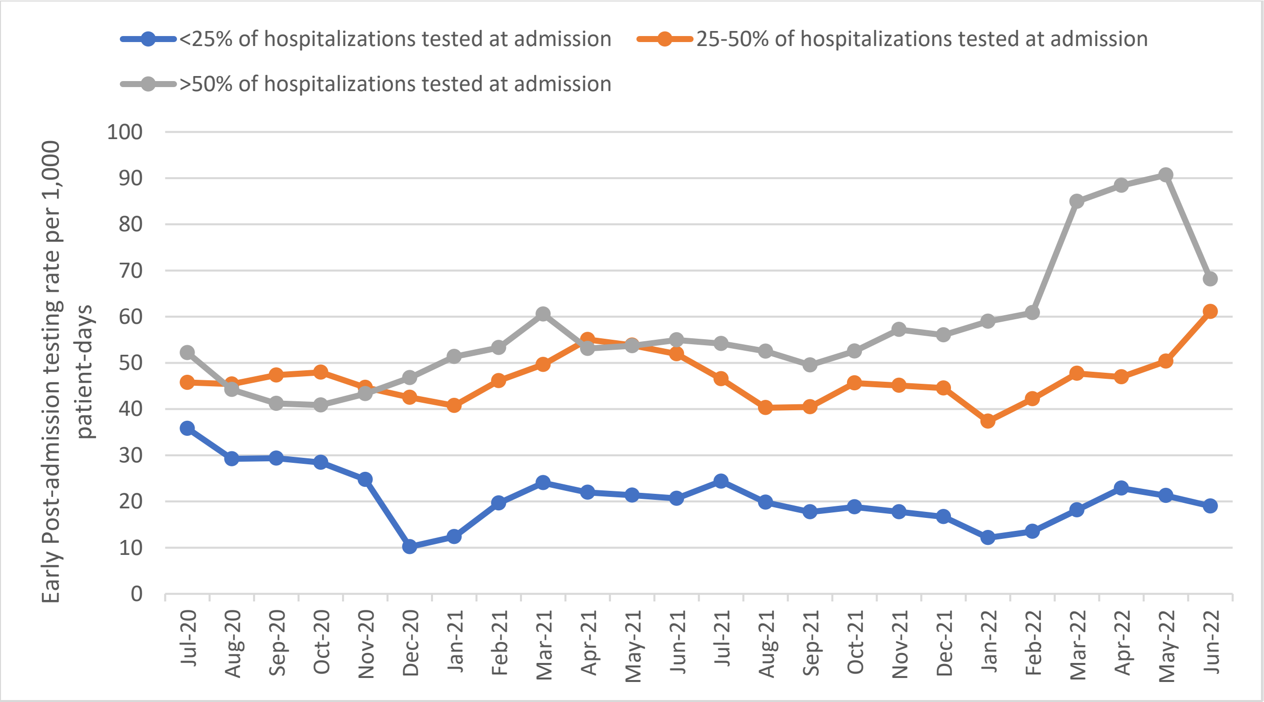

(b) Late post-admission testing (i.e., number of tests after day 7 of admission per 1,000 patient-days)

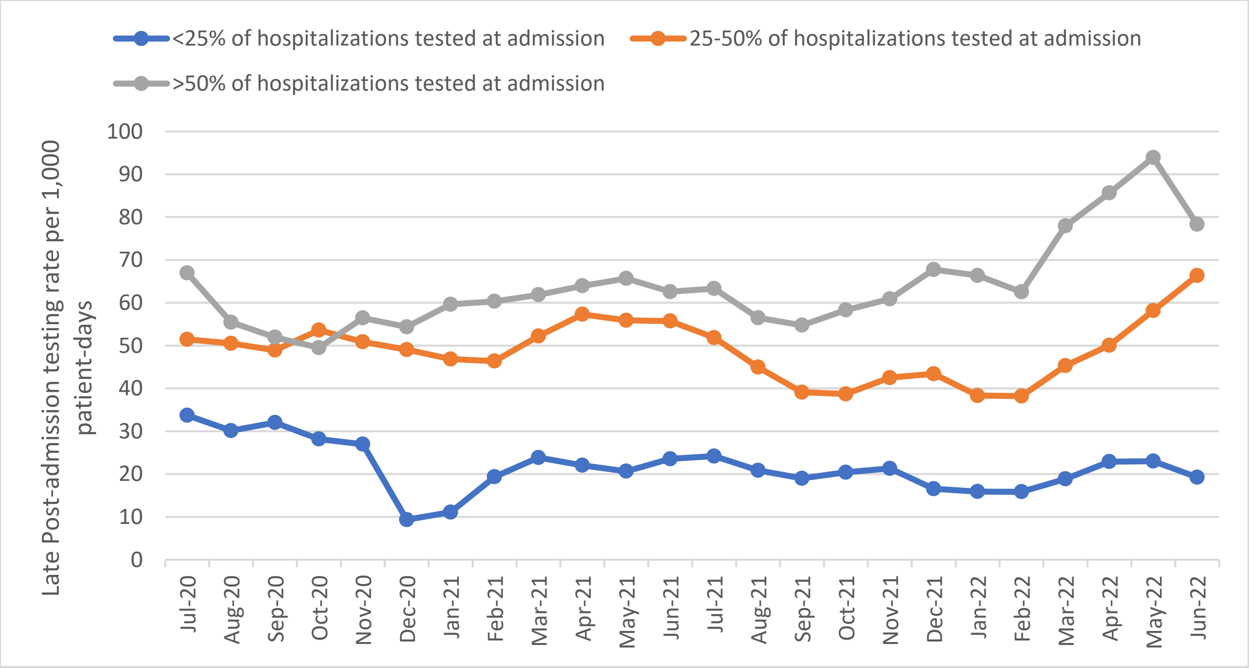

© 2023 Hatfield KM et al. *JAMA Network Open.*

eTable 1. Hospitalization Characteristics of Individuals Stratified by SARS-CoV-2 Infection Status

	No Positive test for SARS-CoV-2 or diagnosis code for COVID-19		Unknown-Onset SARS-CoV-2 Infection		Community-Onset SARS-CoV-2 Infection		Indeterminate-Onset SARS-CoV-2 Infection		Hospital-Onset SARS-CoV-2 Infection	
**Total (Row %)**	4,062,216	95.4%	187,488	4.4%	157,518	3.6%	6,455	0.1%	7,591	0.2%
**Age (Years)**										
< 1	502,515	12.4%	752	0.4%	396	0.3%	13	0.2%	15	0.2%
1 - 17	65,008	1.6%	2,244	1.2%	852	0.5%	36	0.6%	29	0.4%
18 - 44	955,036	23.5%	34,765	18.5%	28,323	18.0%	698	10.8%	714	9.4%
45 - 64	935,537	23.0%	62,674	33.4%	48,798	31.0%	1,684	26.1%	2,310	30.4%
65 - 84	1,281,996	31.6%	70,357	37.5%	62,171	39.5%	2,917	45.2%	3,648	48.1%
85+	322,124	7.9%	16,696	8.9%	16,978	10.8%	1,107	17.1%	875	11.5%
**Race and Ethnicity**										
White, non-Hispanic	2,661,748	65.5%	120,819	64.4%	91,648	58.2%	4,241	65.7%	4,706	62.0%
Black, non-Hispanic	611,294	15.0%	28,889	15.4%	29,502	18.7%	1,062	16.5%	1,436	18.9%
Hispanic	421,415	10.4%	23,104	12.3%	23,347	14.8%	703	10.9%	822	10.8%
Asian, non-Hispanic	105,009	2.6%	3,991	2.1%	3,541	2.2%	99	1.5%	141	1.9%
Other, non-Hispanic	168,589	4.2%	7,521	4.0%	7,151	4.5%	249	3.9%	337	4.4%
Unknown	94,161	2.3%	3,164	1.7%	2,329	1.5%	101	1.6%	149	2.0%
**Sex**										
Female	2,288,856	56.3%	93,071	49.6%	78,537	49.9%	3,252	50.4%	3,356	44.2%
Male	1,773,164	43.7%	94,409	50.4%	78,980	50.1%	3,203	49.6%	4,235	55.8%
**Insurance**										
Medicare	1,761,154	43.4%	93,406	49.8%	83,904	53.3%	4,231	65.5%	4,895	64.5%
Medicaid	862,341	21.2%	24,076	12.8%	21,443	13.6%	706	10.9%	931	12.3%
Commercially Insured	1,126,560	27.7%	55,105	29.4%	38,219	24.3%	1,113	17.2%	1,354	17.8%
Other	312,161	7.7%	14,901	7.9%	13,952	8.9%	405	6.3%	411	5.4%
**Length of Stay,** Median (Q1-Q3)	3	2 - 5	3	5 - 9	5	3 - 10	8	6 - 14	20	14 - 32
**Diagnosis code for COVID-19 infection**	0	0%	187,488	100%	151,076	95.9%	5,573	86.3%	6,779	89.3%
**Tested negative at admission**	1,476,219	36.3%	4,107	2.2%	-	-	2,084	32.3%	2,986	39.3%
**Mechanical Ventilation**	231,202	5.7%	22,349	11.9%	20,550	13.0%	902	14.0%	2,542	33.5%
**Intensive Care Unit Stay**	584,393	14.4%	37,377	19.9%	34,720	22.0%	1,582	24.5%	3,703	48.8%
**Discharge Status**										
Home or self-care	2,683,755	66.1%	104,700	55.8%	82,292	52.2%	1,708	26.5%	1,054	13.9%
Hospice Care	93,243	2.3%	5,815	3.1%	5,519	3.5%	325	5.0%	455	6.0%
Expired	87,548	2.2%	18,835	10.0%	16,964	10.8%	620	9.6%	1,439	19.0%

© 2023 Hatfield KM et al. *JAMA Network Open.*

Other	1,197,670	29.5%	58,138	31.0%	52,743	33.5%	3,802	58.9%	4,643	61.2%
**Time Period**										
Pre-Delta	2,020,260	49.7%	87,337	46.6%	84,215	53.5%	3,519	54.5%	4,249	56.0%
Delta	889,979	21.9%	43,846	23.4%	32,985	20.9%	1,035	16.0%	1,243	16.4%
Omicron	1,151,977	28.4%	56,305	30.0%	40,318	25.6%	1,901	29.5%	2,099	27.7%
**Hospital Census Region**										
Northeast	540,867	13.3%	18,066	9.6%	21,268	13.5%	921	14.3%	1,176	15.5%
Midwest	882,430	21.7%	42,972	22.9%	26,161	16.6%	1,082	16.8%	1,155	15.2%
South	2,450,867	60.3%	119,204	63.6%	104,315	66.2%	4,270	66.2%	4,921	64.8%
West	188,052	4.6%	7,246	3.9%	5,774	3.7%	182	2.8%	339	4.5%

*n=204 observations missing Sex variable

© 2023 Hatfield KM et al. *JAMA Network Open.*

eFigure 2. Aggregate Percent of All Hospitalizations With Community-Onset SARS-CoV-2 Infection, Stratified by the Proportion of Hospitalizations Screened at Admission.

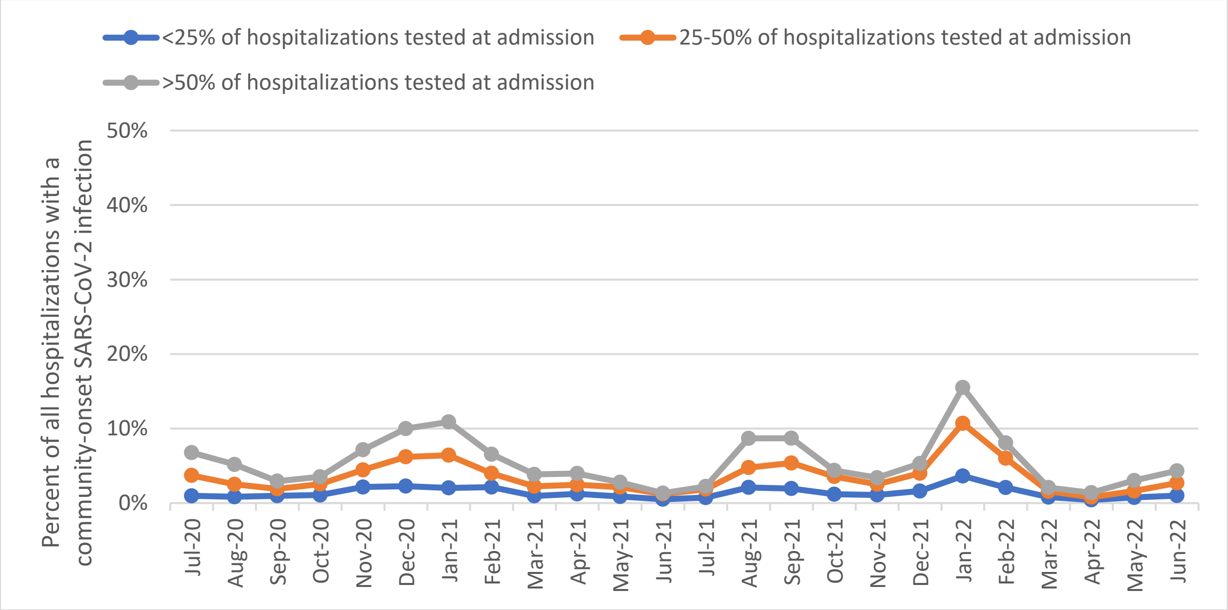

eFigure 3. Aggregate Percent of All Hospitalizations With Unknown-Onset SARS-CoV-2 Infection, Stratified by the Proportion of Hospitalizations Screened at Admission.

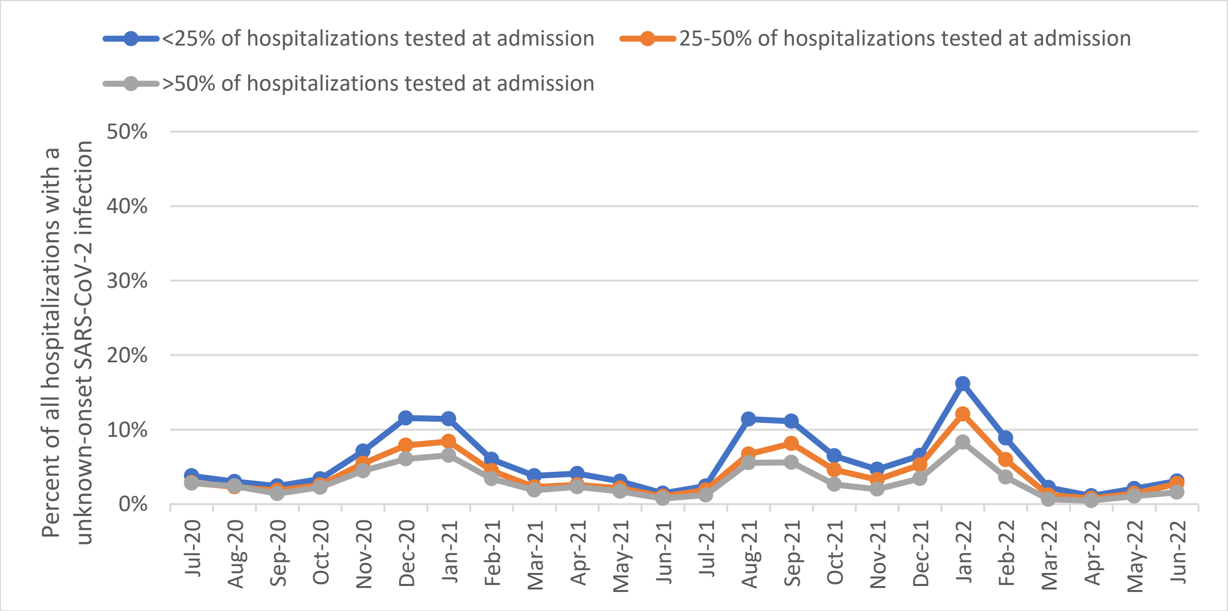

© 2023 Hatfield KM et al. *JAMA Network Open.*

eTable 2. Hospital-Onset SARS-CoV-2 Infections, Stratified by Community-Onset SARS-CoV-2 Infection Rates

Community Onset SARS-CoV-2 Infection Rates	Hospital-months (n)	Hospital-months with at least one hospital-onset SARS-CoV-2 infection (% of hospital-months)	Hospital-onset SARS-CoV-2 Infection Rate, Mean (95% Confidence Interval)			
			Overall	Admissions Tested for SARS-CoV-2		
				<25%	25-50%	>50%
<1%	1,679	274 (16.3)	0.09 (0.05, 0.12)	0.08 (0.04, 0.13)	0.10 (0.07, 0.14)	0.07 (0.02, 0.12)
1 - <2.5%	1,230	528 (42.9)	0.20 (0.18, 0.22)	0.23 (0.19, 0.28)	0.19 (0.16, 0.21)	0.19 (0.14, 0.23)
2.5 - <5%	1,139	586 (51.5)	0.34 (0.30, 0.38)	0.33 (0.25, 0.41)	0.37 (0.31, 0.42)	0.29 (0.22, 0.36)
5% or more	1,639	829 (14.6)	0.48 (0.44, 0.52)	0.38 (0.23, 0.53)	0.52 (0.46, 0.59)	0.46 (0.40, 0.51)
All	5,687	2,217 (39.0)	0.27 (0.26, 0.29)	0.16 (0.13, 0.20)	0.32 (0.30, 0.35)	0.32 (0.29, 0.35)

© 2023 Hatfield KM et al. *JAMA Network Open.*

eFigure 4. Aggregate Hospital-Onset SARS-CoV-2 Infection Rates Using 3 Definitions. Definition 1 includes first positive test after day 7; Definition 2 includes first positive test after day 10; Definition 3 includes first positive test after day 14.

(a)Percent of at-risk hospitalizations (i.e., hospitalizations with a length of stay at least as long as the definition period) with hospital-onset SARS-CoV-2 infections

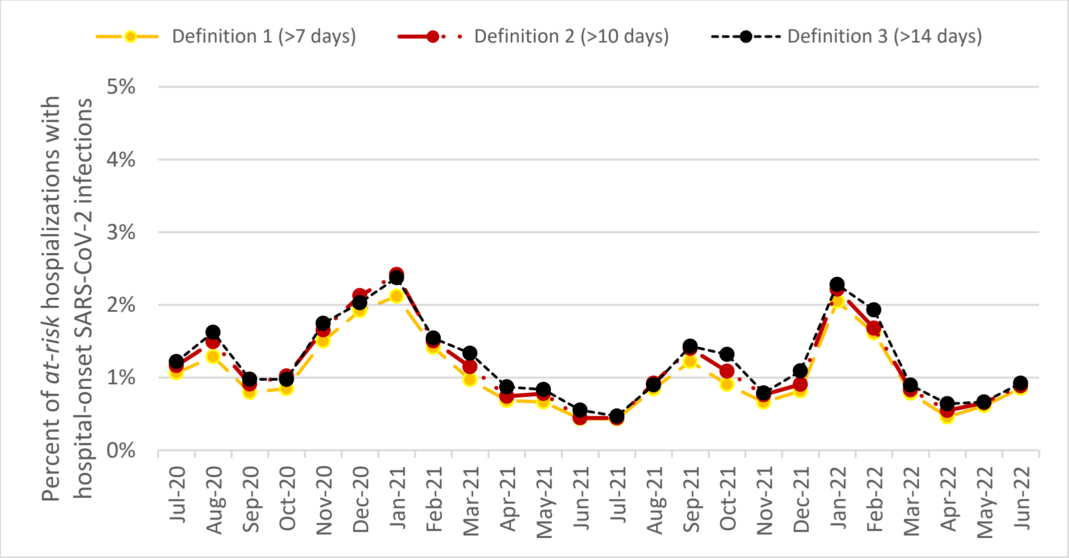

(b) Percent of all hospitalizations with hospital-onset SARS-CoV-2 infections

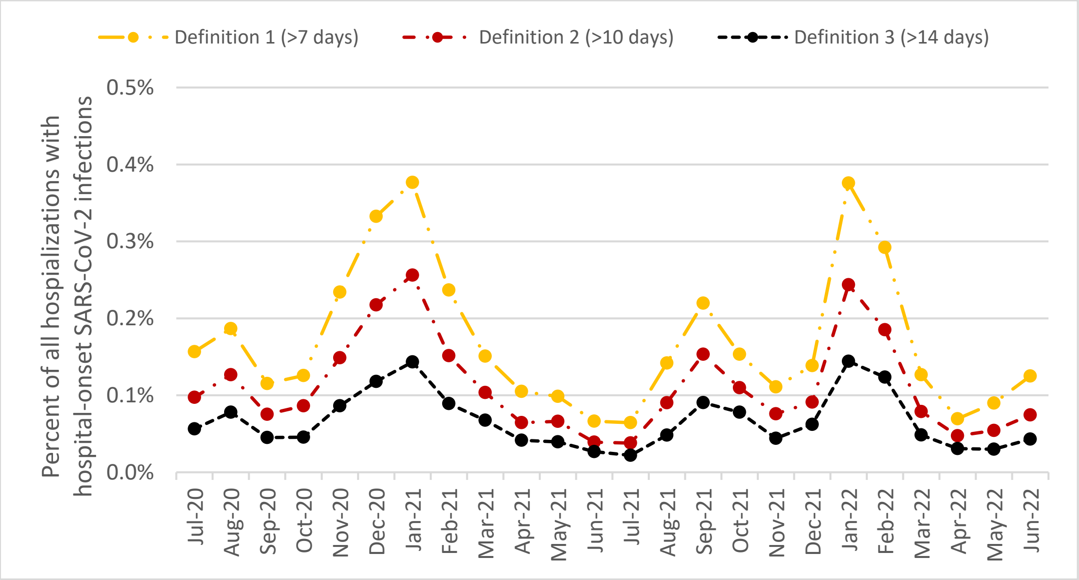

© 2023 Hatfield KM et al. *JAMA Network Open.*

(c) Rate of hospital-onset SARS-CoV-2 infections per 1,000 patient days

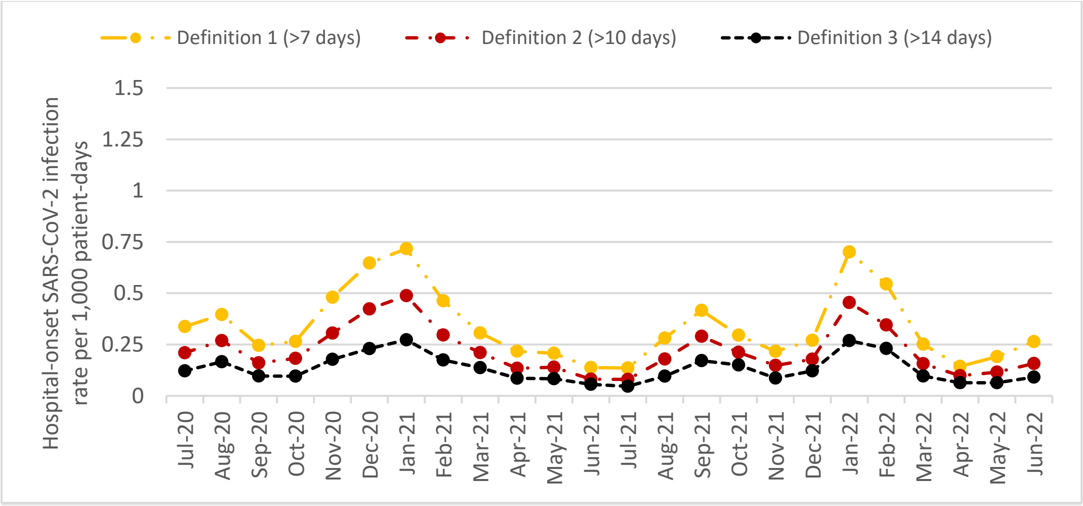

eFigure 5. Aggregate Hospital-Onset SARS-CoV-2 Infection Rates per 1000 Patient-Days Stratified by the Proportion of Hospitalizations Screened at Admission.

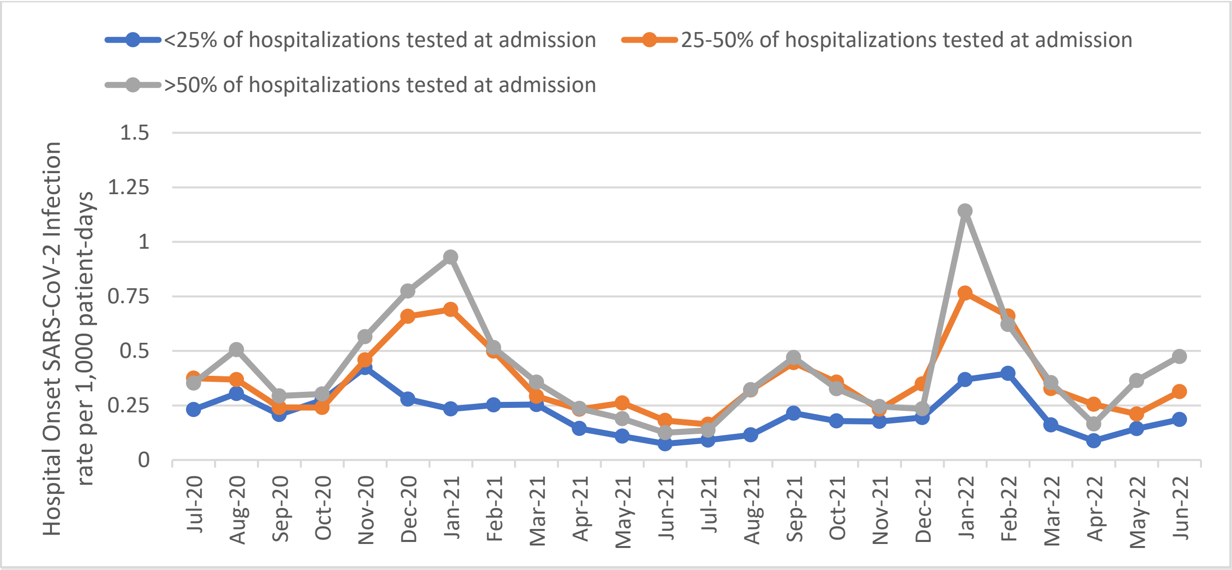

© 2023 Hatfield KM et al. *JAMA Network Open.*
