# Supplementary material for: Assessment of Hospital-Onset SARS-CoV-2 Infection Rates and Testing Practices in the US, 2020-2022
Source: JAMA Netw Open. 2023 Aug 28;6(8):e2329441. doi: 10.1001/jamanetworkopen.2023.29441 (PMC10463096; doi:10.1001/jamanetworkopen.2023.29441)
Supplement: Supplement 2. — Data Sharing Statement [file jamanetwopen-e2329441-s002.pdf]

Data Sharing Statement

Hatfield. Assessment of Hospital-Onset SARS-CoV-2 Infection Rates and Testing Practices in the US. *JAMA Netw Open*. Published August 28, 2023.
doi:10.1001/jamanetworkopen.2023.29441

Data
Data available: No

Additional Information
Explanation for why data not available: Our analysis is based off proprietary datasets from PINC AI™ Applied Sciences Healthcare Data (see: https://offers.pinc-ai.com/PINC-AI-Healthcare-Database-White-Paper-LP.html).
